# Metastatic endophthalmitis combined with subretinal abscess in a patient with diabetes mellitus—a case report

**DOI:** 10.1186/s12886-015-0079-y

**Published:** 2015-08-14

**Authors:** Tsung-Han Tsai, Kai-Ling Peng

**Affiliations:** Department of Ophthalmology, Chi Mei Medical Center, No.901, Zhonghua Rd., Yongkang Dist., Tainan City, 710 Taiwan

**Keywords:** Metastatic endophthalmitis, Subretinal abscess, Vitrectomy with intravitreal injection of antibiotics

## Abstract

**Background:**

Endogenous endophthalmitis, extra-hepatic metastasis from liver abscess with diabetes mellitus, could lead to a devastating outcome without a prompt and appropriate management. We report a case of metastatic endophthalmitis combined with subretinal abscess with successful visual outcome after treatment.

**Case presentation:**

A 56-year-old male patient with diabetes mellitus under poor control presented to our emergency room with fever, sore throat, cough and poor appetite for 2 weeks. Abdominal computed tomography showed a 2.2 × 2.0 cm liver abscess. During hospitalization, sudden onset of blurred vision with floaters in his left eye was noted.

Meanwhile, Brain computed tomography demonstrated subdural abscess in right parietal area. With obvious vitritis, a localized subretinal abscess was also found over temporal arcade with size about four disc areas under indirect ophthalmoscopy. A pars plana vitrectomy with intravitreal injection of ceftazidime (2 mg/0.1 ml) and amikacin (0.4 mg/0.1 ml) was performed without retinectomy. The margin of the subretinal abscess became firm and the central area resolved after the operation. Finally, his vision improved to 6/6 after cataract surgery.

**Conclusions:**

Subretinal abscess is an extremely rare presentation of metastatic endophthalmitis. It is difficult to develop appropriate treatment guidelines of endophthalmitis complicated with subretinal abscess. Our experience in this case demonstrated if the size of the subretinal abscess is smaller than four disc areas, pars plana vitrectomy with intravitreal injection of antibiotics without retinectomy could be considered to avoid further retinal detachment.

## Background

Subretinal abscess, a solitary and yellowish-white circumscribed lesion with hemorrhages in the overlying retina in the posterior fundus, is an extremely rare presentation of endogenous bacterial endophthalmitis (EBE), which accounts for approximately 5 % of endophthalmitis cases [1, 2]. Endogenous endophthalmitis, also termed metastatic endophthalmitis, occurs when organisms from other infection foci enter the internal ocular spaces by crossing the blood-ocular barrier. These organisms travel via circulation to the choroid, Bruch membrane and the retinal pigment epithelium [3], which end up with the accumulation of subretinal abscess. Due to its rarity, it is difficult to develop appropriate treatment guidelines of endophthalmitis combined with subretinal abscess.

We reported a rare case of metastatic endophthalmitis combined with subretinal abscess in a diabetic male patient with liver and subdural abscess. His vision successfully improved after vitrectomy with intravitreal injection of antibiotics.

## Case presentation

A 56-year-old male patient with medical history of type 2 diabetes mellitus for about 8 years under oral medication at local clinic presented with fever, poor appetite and general weakness for 2 weeks. Hyperglycemia and leukocytosis were noted in emergency room. Abdominal computed tomography was arranged due to right upper abdominal pain, which showed a liver abscess with size of 2.2 × 2.0 cm. He was admitted for intravenous injection of cefuroxime (1.5gm q8h) and further evaluation.

**Fig. 1:**
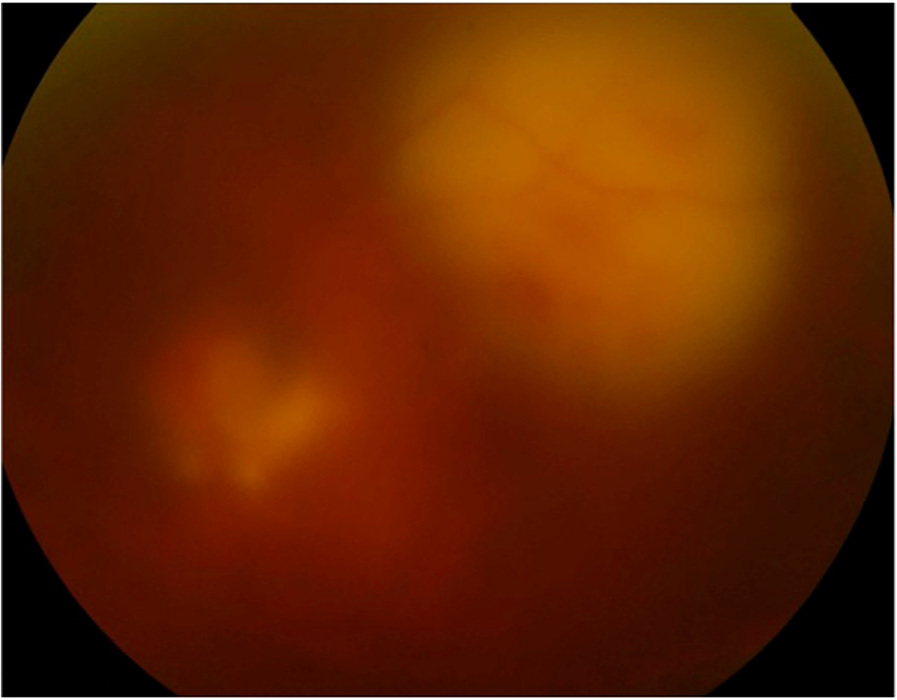
Obvious vitritis and two localized subretinal abscess were noted with irregular surfaces and fluffy margins. The larger one was located over temporal arcade and about four disc areas large. The smaller one was just at the lower disc margin

**Fig. 2:**
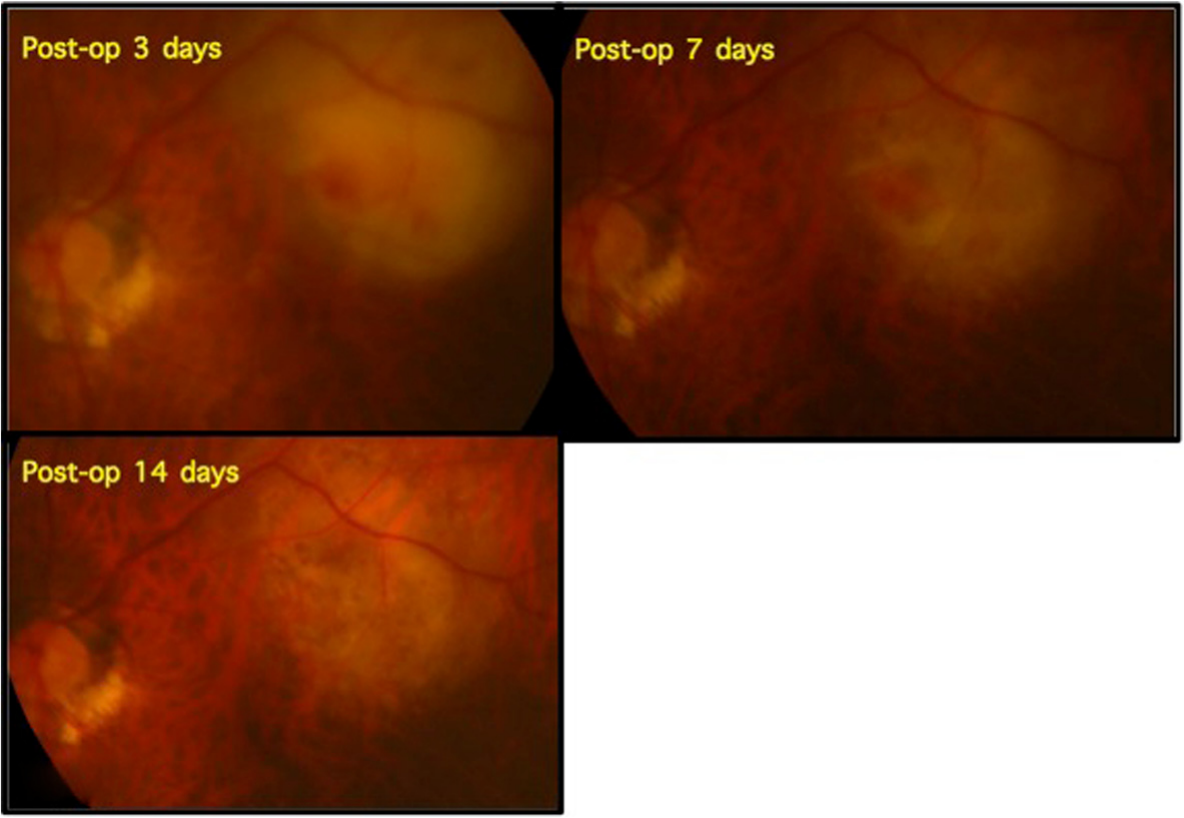
The margin of the subretinal abscess became firmed and the central part resolved later. The area of previous subretinal abscess gradually developed into retinal pigment epithelium mottling without fovea involvement

**Fig. 3:**
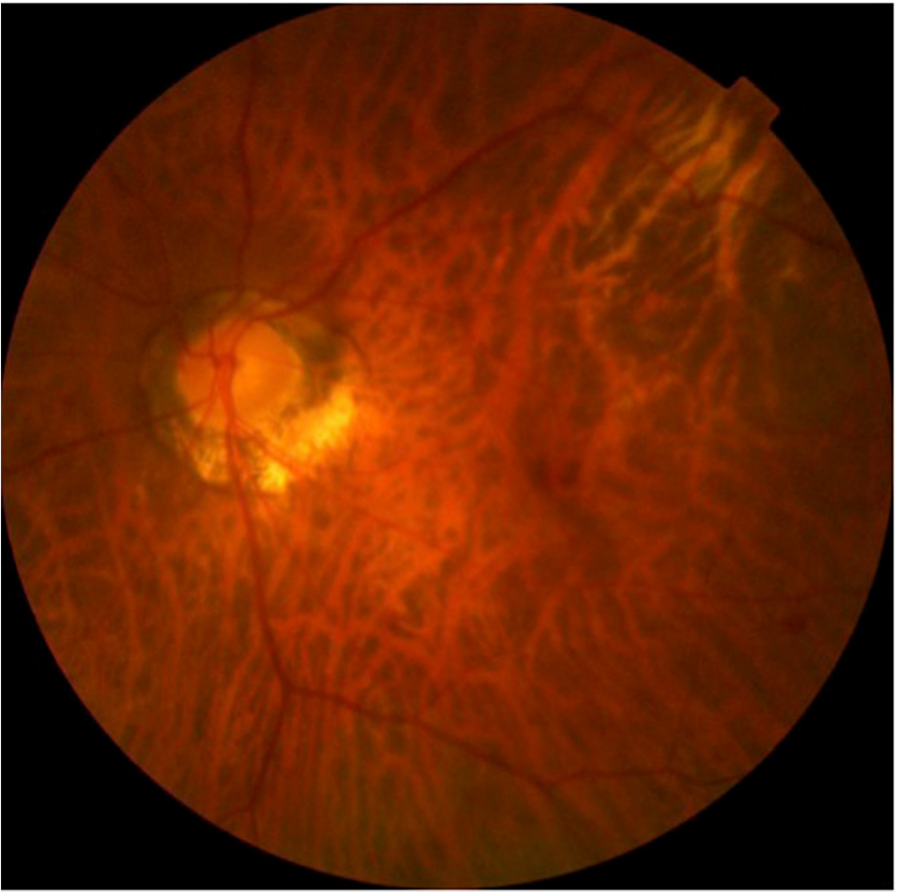
The picture of 1 year after surgery showed no fovea involvement

The patient presented sudden onset blurred vision of left eye with floaters on the 4th day of hospitalization. Obvious vitritis was found under indirect ophthalmoscopy. Two localized subretinal abscess were noted with irregular surfaces and fluffy margins. The larger one was located over temporal arcade with size about four disc areas. The smaller one was just at the lower disc margin (Fig. 1). Meanwhile, left upper limb involuntary movement and focal seizure were also noted. Brain computed tomography revealed right subdural abscess. We performed a pars plana vitrectomy with intravitreal injection of ceftazidime (2 mg/0.1 ml) and amikacin (0.4 mg/0.1 ml) without retinectomy on the 5th day of hospitalization. Fortunately, the margin of the subretinal abscess became firm and the central part resolved later. At last, the area of previous subretinal abscess gradually developed into retinal pigment epithelium mottling without fovea involvement (Fig. 2). He received craniotomy later due to progression of subdural abscess under intravenous antibiotics of ceftriaxone (2gm q12h). The culture results of blood, vitreous and subdural abscess were all negative. His vision in left eye improved to 6/6 after cataract surgery 1 year after the episode (Fig. 3).

## Conclusion

The prevalence of EBE could be as high as 90 % in patients with diabetes, cardiac disease and malignancy. According to the major review of Timothy L. Jacksonand associates, 56 % of 267 patients had comorbidities that predisposed to infection. The most common condition was diabetes, particularly in association with klebsiella liver abscess [4].

Patients presented with systemic infections such as liver abscess, meningitis, and endocarditis may subsequently develop EBE.

Cheng and associates conducted a retrospective search for 23 septic metastatic lesions of 187 consecutive patients with pyogenic liver abscess that there were 60.8 % of endophthalmitis, 43.4 % of pulmonary abscess, 26 % of brain abscess or purulent meningitis, 21.7 % bacteriuria or prostatic abscess and 8.6 % of osteomyelitis and pyogenic arthritis [5].

Our case was a male diabetic patient with poor glycemic control whose septic metastatic lesions were subdural abscess, endogenous endophthalmitis with subretinal abscess and liver abscess.

The presentations of fundus with endophthalmitis may be nonspecific such as vitritis, retinal hemorrhages, nerve fiber layer infarction, retinitis, perivasculitis, and subretinal exudation. Manifestation such as subretinal abscess was quite rare. The most common bacterial subretinal abscess which caused by Norcardia is seen in patients with underlying immunosuprression [6, 7]. Other causes are rare. There were three individual case reports demonstrated involving Pseudomonas aeruginosa [8], Streptococcus Viridans [9], and pneumoniae [10]. The vision of those cases after treatment was variable. However, the prognosis of most cases was poor. Eddie W. Harris and associates [11] reported a case of klebsiella endophthalmitis with subretinal abscess promptly intervened with extensive retinectomy, complete abscess excision and intravitreal antibiotic therapy resulted in relatively better vision than previous cases.

In our case, we performed pars plana vitrectomy with intravitreal injection of antibiotics of ceftazidime (2 mg/0.1 ml) and amikacin (0.4 mg/0.1 ml) without retinectomy considering the limited size of subretinal abscess (four disc areas). We observed that the margin of subretinal abscess became well demarcated and the central part firmed gradually after surgery. The subretinal abscess resolved, only mottled retinal pigment epithelium left.

The modality of treatment and visual prognosis of subretinal abscess combined with endogenous endophthalmitis depends on its severity, such as turbidity of vitritis, location and extent of subretinal abscess and retinal exudation. Theoretically, advantages of vitrectomy include removal of the infection organisms, endotoxins and vitreous membranes that could lead to retinal detachment; clearing of vitreous opacities; collection of abundant material for culture and possibly better distribution of intravitreal antibiotics. The more extensive the surgery is, the more exudation and inflammatory substance would accumulate on the retina and in the vitreous cavity, which may interfere with the identification between infection progression and inflammation flares up. However, if subretinal abscess is really large and high, vitrectomy combined with retinectomy to remove adequate abscess should be considered to decrease the amount of bacteria and to facilitate infiltration of antibiotics. Intravitreal injection of antibiotics combined with appropriate amount of steroids should also be considered after surgery to decrease surgery-related inflammation.

Subretinal abscess is an extremely rare presentation of metastatic endophthalmitis. It is difficult to develop appropriate treatment guidelines of endophthalmitis complicated by subretinal abscess. Our experience in this case demonstrated if the size of the subretinal abscess is smaller than four disc areas, pars plana vitrectomy with intravitreal injection of antibiotics without retinectomy could be considered to avoid further retinal detachment.

## Consent

Written informed consent was obtained from the patient for publication of this Case Report and accompanying images. A copy of the written consent is available for review by the Editor of this journal.

## Abbreviations

EBE: Endogenous bacterial endophthalmitis
